# *Agrobacterium fabrum* *atu0526*-Encoding Protein Is the Only Chemoreceptor That Regulates Chemoattraction toward the Broad Antibacterial Agent Formic Acid

**DOI:** 10.3390/biology10121345

**Published:** 2021-12-17

**Authors:** Hao Wang, Mengqi Zhang, Yujuan Xu, Renjie Zong, Nan Xu, Minliang Guo

**Affiliations:** Department of Biotechnology, College of Bioscience and Biotechnology, Yangzhou University, Yangzhou 225009, China; wanghao@yzu.edu.cn (H.W.); ZMQi68@163.com (M.Z.); xuyujuan18352768038@outlook.com (Y.X.); rjiezong@163.com (R.Z.); nanxu@yzu.edu.cn (N.X.)

**Keywords:** methyl-accepting chemotaxis protein, *Agrobacterium fabrum*, chemotaxis, formic acid chemoreceptor, protein ligand

## Abstract

**Simple Summary:**

Soil-borne plant pathogens generally navigate their way to hosts through recognition of the root exudates by chemoreceptors. However, there is still a lack of appropriate identification of chemoreceptors and their ligands in the typical soil-borne plant pathogen *Agrobacterium*. Here, we characterize Atu0526 as a Cache-type chemoreceptor from *Agrobacterium fabrum* C58, identify the potential ligands interacting with Atu0526 and analyze the possible signal transduction mechanism after ligand binding. We confirm Atu0526 to be the receptor of the broad antibacterial agent formic acid. The deletion of *atu0526* completely abolished the chemotaxis of *A. fabrum* toward formic acid. Further experiments showed that the residue Arg 115 plays an essential role in the chemotactic function. Molecular modelling suggests that Arg 115 provides “an anchorage” for formic acid to pull the minor loop, thereby forming a conformational change that may be essential for signal transduction. Identifying the first chemoreceptor of antimicrobial agent formic acid in *Agrobacterium* will provide new perspectives on bacteria navigating their way to the hosts, and the discovered key arginine site can significantly improve our understanding of the signal transduction mechanism of single-Cache-type chemoreceptors.

**Abstract:**

Soil-born plant pathogens, especially *Agrobacterium*, generally navigate their way to hosts through recognition of the root exudates by chemoreceptors. However, there is still a lack of appropriate identification of chemoreceptors and their ligands in *Agrobacterium*. Here, Atu0526, a sCache-type chemoreceptor from *Agrobacterium fabrum* C58, was confirmed as the receptor of a broad antibacterial agent, formic acid. The binding of formic acid to Atu0526 was screened using a thermo shift assay and verified using isothermal titration calorimetry. Inconsistent with the previously reported antimicrobial properties, formic acid was confirmed to be a chemoattractant to *A. fabrum* and could promote its growth. The chemotaxis of *A. fabrum* C58 toward formic acid was completely lost with the knock-out of *atu0526*, and regained with the complementation of the gene, indicating that Atu0526 is the only chemoreceptor for formic acid in *A. fabrum* C58. The affinity of formic acid to Atu0526^LBD^ significantly increased after the arginine at position 115 was replaced by alanine. However, in vivo experiments showed that the R115A mutation fully abolished the chemotaxis of *A. fabrum* toward formic acid. Molecular docking based on a predicted 3D structure of Atu0526 suggested that the arginine may provide “an anchorage” for formic acid to pull the minor loop, thereby forming a conformational change that generates the ligand-binding signal. Collectively, our findings will promote an understanding of sCache-type chemoreceptors and their signal transduction mechanism.

## 1. Introduction

*Agrobacterium fabrum* is a soil-borne Gram-negative bacterium that infects a variety of dicotyledonous plants. Plants secrete organic acids and other compounds near the rhizosphere, thus forming an acidic environment [1,2,3]. Sensing rhizospheric secretions through chemotaxis is the first step for *A. fabrum* to navigate its way to the host plant [4,5,6]. Chemotaxis permits bacteria to perceive their external surroundings (chemicals, pH, redox potential, temperature, etc.) and to swim toward a favorable environment [7,8,9]. Chemoreceptors and the histidine kinase play essential roles in chemotactic signaling [10,11]. Methyl-accepting chemotaxis proteins (MCPs) are the most common chemoreceptors of various ligands (attractants and repellents) [12]. Ligand binding induces conformational changes in MCP molecules, shifting the ON-OFF equilibration of CheA auto-phosphorylation activity [13]. CheY is thereby phosphorylated, subsequently triggering a rotation adjustment of the flagellar [14]. The signal transduction also requires a coupling protein, CheW. CheW couples CheA to the MCP dimers to form a ternary core complex [15,16,17]. This interaction between CheWs and MCPs is necessary for chemotactic signaling [16].

MCPs, widely existing in bacteria, normally contain three functional elements, including a ligand-binding domain (LBD), an intervening HAMP domain and a cytoplasmic methyl-accepting (MA) signaling domain [12]. For the MCP with periplasmic LBD, its LBD is linked to the intervening HAMP domain via transmembrane domains. The ligand-binding signal is generated by the LBD and transmitted through HAMP to the MA signaling domain [13]. In general, the HAMP domain and the MA signaling domain are evolutionarily conserved, while LBDs display complicated diversity for the perception of various ligands and signals [18].

*Agrobacterium* LBDs are distributed among six different types, including single 4HB (four-helix bundle), double 4HB, single Cache (single calcium channels and chemotaxis, sCache), double Cache (dCache), PAS (Per/Arnt/Sim) and protoglobin [19]. Cache-type MCPs account for the largest proportion of chemoreceptors in *Agrobacterium* [19]. The Cache domain was initially considered to be an extracellular PAS-like domain until more in-depth analysis and detailed classification appeared [20]. A dCache domain is composed of two subdomains, each homologous to the PAS domain, and a long N-terminal α-helix [21], whereas a sCache domain has only one “PAS-like” domain and a relatively short N-terminal α-helix. sCache is further divided into sCache_2, sCache_3_1, sCache_3_2 and sCache_3_3. The known ligands of all four sCache subfamilies are mainly organic acids [20]. In *A. fabrum* C58, there are seven MCP candidates belonging to the Cache superfamily, including Atu0373, Atu0526, Atu0646, Atu1912, Atu2173, Atu2223 and Atu3725 [19]. Little research has been devoted to these proteins.

Generally, the ligand-binding signal is generated by a conformational change of the LBD. The Apo- and ligand-bound 4HB-type LBD structures showed a certain degree of conformational changes [22,23,24]. Several 4HB-type LBDs undergo an approximately 1 Å piston-like shift of the final helix (H4) when ligands bind [25,26]. It is proposed that the ligand binding to Cache domains may also trigger conformational changes, likely by piston displacement, although the structures of Cache-type LBDs are distinct from 4HB-type LBDs [27]. The structural analysis of Tlp3, a dCache-type MCP from *Campylobacter jejuni*, suggested that the binding of an attractant to the distal subdomain locks it in a closed form, altering the proximal subdomain into an open form, which results in a 4 Å piston displacement of the C-terminal helix [28]. For the sCache-type MCP, there is still a lack of more convincing evidence to reveal the related signal generation mechanism.

Formic acid has been used widely and long-term as a poultry feed additive to inhibit the growth of foodborne pathogens [29]. The treatment of *E. coli* with formic acid significantly reduced the synthesis rate of its DNA, RNA, protein, phospholipids and cell wall [30]. Since formic acid is considered to be an antibacterial agent, evolutionarily, it is likely to act as a chemorepellent for bacteria. However, some studies contradict this hypothesis. For instance, formic acid was confirmed to be a chemoattractant for *C. concisus* and *C. jejuni* [31,32]. To date, no studies have been conducted on the chemotactic response of *A. fabrum* to formic acid.

Currently, the studies of chemoreceptors for formic acid are still limited. Two adjacent genes, *cj0952c* and *cj0951c*, from *C. jejuni* isolate B2, have been found to affect the chemotactic behavior of the strain toward formic acid [33]. However, there is no evidence that the proteins encoded by these two genes are the chemoreceptor for formic acid. Thus far, the only confirmed chemoreceptor that binds formic acid is McpV from *Sinorhizobium meliloti* [34]. However, the dissociation constant (*K_d_*) of McpV^PR^ (the LBD of McpV) binding formic acid is much higher than the *K_d_* of McpV^PR^ binding some other short-chain carboxylates. The optimal concentration of formic acid causing the chemotactic response of *S. meliloti* is 100 mM, which is much higher than the concentration of formic acid occurring in natural conditions [34]. These data indicate that formic acid interacts weakly with McpV and is an inefficient chemoattractant to *S. meliloti* [34].

Here, we characterized the first formic acid specific chemoreceptor and analyzed its possible signal transduction mechanism.

## 2. Materials and Methods

### 2.1. Strains and Their Growth Conditions

The strains and plasmids used in this study are listed in Supplementary Materials Table S1. *E. coli* was cultured at 37 °C in Luria Broth (LB) medium with or without 1.5% agar [35]. *A. fabrum* was cultured at 28 °C in MG/L (0.5% *w/v* tryptone, 0.25% *w/v* yeast extract, 90 mM NaCl, 55 mM D-mannitol, 12.4 mM sodium glutamate, 3.7 mM KH_2_PO_4_, 0.8 mM MgSO_4_, 8 nM D-biotin, pH 7) or AB-sucrose (0.5% *w/v* sucrose, 18.7 mM NH_4_Cl, 17.2 mM K_2_HPO_4_, 9.6 mM NaH_2_PO_4_, 2 mM KCl, 1.2 mM MgSO_4_, 90 μM CaCl_2_, pH 7) medium with or without 1.5% agar [36,37]. For *E. coli* transformed with antibiotic resistance, 100 µg/mL ampicillin or 50 µg/mL kanamycin was used, while for *A. fabrum*, 100 µg/mL kanamycin or 100 µg/mL carbenicillin was used.

### 2.2. Gene Manipulation, Construction of Mutants and Complementary Strains

Genomic DNA of *A. fabrum* was prepared according to a previous study [37]. Plasmids were extracted and purified with TIANprep Mini Plasmids Kit (Tiangen Biotech Corporation, Beijing, China). The primers used in this study are listed in Supplementary Materials Table S2. DNA fragments were purified from agarose gels using a TaKaRa MiniBEST Agarose Gel DNA Extraction Kit (TaKaRa Corporation, Dalian, China). Plasmids were transformed into *E. coli* competent cells through heat-shocking at 42 °C. Plasmids were transformed into *A. fabrum* through electroporation [38].

The entire *atu0526* gene was knocked out using the method described previously [39]. pEX18Km, which carries a kanamycin resistant gene for a positive selection and a suicide gene, *sacB,* for counter-selection, was used for constructing a gene-knockout mutant [40]. The fragments of *atu0526* upstream (500 bp fragment upstream of start codon) and downstream (500 bp fragment downstream of stop codon) were fused and inserted into pEX18Km though restriction sites *Hin*d III and *Bam*H I. After this construct was transformed into *A. fabrum*, two rounds of selections were performed using kanamycin and sucrose to obtain knockout candidate colonies. The colonies were further screened using a PCR and verified using DNA sequencing.

A complementary strain was obtained by introducing the plasmid carrying *atu0526* with its promoter into the knockout strain. In brief, the DNA fragment of *atu0526* with its promoter was amplified using the primers *atu0526*C-F and *atu0526*C-R listed in Supplementary Materials Table S2. The fragment was then inserted into the independently replicating plasmid pCB301 with a low copy number though restriction sites *Hin*d III and *Bam*H I. The construct was subsequently transformed into the *atu0526*-knockout strain. The complementary colonies were selected by kanamycin and verified using PCR and DNA sequencing.

The single-residue mutation was carried out as described previously [41]. In brief, a pair of single-residue mutation primers (R115A-F and R115A-R) were designed around the position of 115 (amino acid number of Atu0526). The *atu0526* complementary plasmid was amplified with single-residue mutation primers using a PCR. The template plasmid was digested for removal using *Dpn* I. Then, the product was purified and transformed into *E. coli* DH5α competent cells. The constructed plasmid was extracted from the positive transformants, and the mutation was verified using DNA sequencing. The verified plasmid was then transformed into the *atu0526*-knockout strain by electroporation. The resulted colonies were screened by kanamycin and verified using PCR.

### 2.3. Capillary Assay

The capillary assay was carried out as described previously [42]. *A. fabrum* in logarithmic growth phase was harvested by centrifugation at 4000× *g* for 2 min at room temperature and then suspended in chemotaxis buffer (0.1 mM EDTA and 10 mM KH_2_PO_4_, pH 7.0) to an OD_600nm_ of 0.1. Every aliquot of 300 μL of suspension was used to make 1 bacterial pond. The capillary tube with one end sealed was filled with chemotaxis buffer containing 500 μM neutralized formic acid or no formic acid. The open end of the capillary tube was put into the bacterial pond and incubated for 1 h at room temperature. The resulting solution in the capillary tube was completely transferred into 1 mL of AB-sucrose medium. An amount of 10 μL of diluted solution was plated on MG/L plates and incubated for 2 days at 28 °C. The colonies on the plates were finally counted and analyzed.

### 2.4. Protein Expression and Purification

The periplasmic domain of Atu0526 (from 36 th residue to 197 th residue) was expressed using pET30a. The corresponding gene fragment was inserted into pET30a and then transformed into *E. coli* BL21 (DE3) competent cells. An empty pET30a was also transformed into BL21 to express a control protein since the empty pET30a can also express a 7.7 kDa His-tagged peptide. Positive transformants were grown to approximately 5 × 10^8^ cell/mL in LB medium at 37 °C. Then, 0.5 mM isopropylthio-β-D-galactoside was added to the cultures. After a 5-h cultivation at 100 rpm and 25 °C, cells were harvested by centrifugation of 8000× *g* for 10 min, and then washed twice with PBS buffer (10 mM phosphate, pH 7.4). Cell suspension was subsequently sonicated until it was nearly clear, and cell debris was removed by centrifugation (12,000× *g* for 30 min). The supernatant was slowly mixed with 1.5 mLof ProteinIos Ni-IDA Resins (Transgen Biotech, Beijing, China). His-tagged proteins were eluted from the Ni-IDA Resins with elution buffer (150 mM NaCl, 200 mM imidazole, 50 mM Tris-HCl pH 7.4). The imidazole was removed by ultrafiltration.

### 2.5. Pull-Down Assay and Bacterial Two-Hybrid Assay

The pull-down assay was carried out as described previously [42]. His-tagged Atu0526 was expressed as described above. Another His-tag fused protein, SalT, a NaCl induced protein encoded by *atu0661*, which is unrelated to the chemotaxis, was used as the negative control [42]. The *E. coli* BL21 crude lysates containing the His-tagged Atu0526 or SalT were incubated with cell crude extract from 100 mL of *Δatu0526* cell culture. After overnight incubation at 4 °C with gentle shaking, the resins were washed with 10 resin volumes of PBS buffer containing series concentrations of imidazole (10–50 mM) to remove the non-specific binding proteins. Target proteins were eluted with 500 µL of PBS buffer containing 200 mM imidazole. Equal volumes (10 µL) of elutions were loaded for SDS-PAGE. Proteins in the gel were transferred to polyvinylidene difluoride membranes (Merck Millipore, Darmstadt, Germany) and were detected with the BCIP/NBT alkaline phosphatase color development kit (Beyotime Biotechnology Corp, Shanghai, China) according to the procedure provided by the company. Antibodies against two CheWs were used as the primary antibodies for detection [42]. Two polyclonal rabbit antibodies against CheW1 and CheW2 were provided by GenScript Corporation (Nanjing, China). Peptides from the variable region of two CheWs (142–155 amino acid residues of CheW1, 146–159 amino acid residues of CheW2) were artificially synthesized as the antigens to generate antibodies against CheW1 or CheW2 in New Zealand rabbits. The specificities of the antibodies against each CheW had already been verified by the heterogeneously produced individual CheW using Western blot in a previous study [42].

The bacterial two-hybrid assay was conducted as previously described [43]. *atu0526* was inserted into the bait plasmid pBT to express λcI-Atu0526 fusion protein. The entire *cheW* genes were inserted into the target plasmid pTRG to express CheW–RNAP fusion proteins. The combinations of the bait and the target were then transformed into XL1-Blue MR competent cells. Transformed cells were spread on LB-CTCK plates (LB with 0.2 mg/mL carbenicillin, 15 μg/mL tetracycline, 34 μg/mL chloramphenicol and 50 μg/mL kanamycin). Positive colonies were spotted on LB-CTCK plates with 80 μg/mL X-GAL and incubated in darkness for 17 h at 37 °C. The interaction between Atu0526 and CheWs would carry λcI and RNAP together, thereby inducing the expression of β-galactosidase. The bacterial colonies would be blue when grown on plates containing 80 μg/mL X-GAL.

### 2.6. Thermo Shift Assay

Thermo shift assay was carried out as described previously [44,45]. In brief, each purified protein was mixed with or without 500 μM formic acid in the presence of SYPRO Orange (Thermo Fisher Scientific, Waltham, MA, USA), which was buffered by 50 mM Tris-HCl (pH 7.4). Mixtures were then heated from 25 to 95 °C at a scan rate of 0.5 °C per 30 s using a Real Time PCR instrument (BioRad, Hercules, CA, USA). Unfolding curves of proteins were monitored by detecting changes of fluorescent intensity. Melting temperatures were determined from the derivative values of fluorescence data using BioRad CFX Manager 3.1 software.

### 2.7. Isothermal Titration Calorimetry

Isothermal titration calorimetry was conducted on a VP microcalorimeter (Malvern Panalytical, Malvern, UK) at 20 °C. Purified proteins, including control protein expressed from empty pET30a, were concentrated to 30 μM in sample buffer (150 mM NaCl, 50 mM Tris-HCl pH 7.4) and injected into the sample cell. The proteins were titrated with sample buffer containing 0.6 mM formic acid. The mean enthalpies measured from the injection of the ligand into the sample cell were subtracted from the data of control protein titration prior to data analysis. Data points were fitted with the “one binding site model” of ORIGIN to calculate *K_d_* and other values.

### 2.8. Fluorescence Observation and Analysis

For microscopy observation, the *Agrobacterium* cells in the middle logarithmic growth period were collected by centrifugation at 4000× *g* for 3 min and then suspended in PBS buffer. The bacterial solutions were added onto the center of the slides. Cells were visualized through a ×100 oil immersion objective of a fluorescent microscope (Mshot Corp., Guangzhou, China). Fluorescence was observed using an Ar laser with a 488-nanometer excitation wavelength and 500~550-nanometer emission wavelength.

### 2.9. Analysis of Biofilm Formation

C58, *△atu0526*, *△atu0526*-C and *△CheA* (since *△atu0526*-C contains the plasmid pCB301::*atu0526* carrying a kanamycin resistant gene, we introduced the empty vector pCB301 into C58, *△atu0526* and *△CheA*) were grown in liquid MG/L with 100 µg/mL kanamycin at 28 °C, 200 rpm for 14 h. After washing twice with AB-sucrose liquid medium, the concentration of cells was adjusted to OD_600_ = 1.0. Then the resuspensions were diluted 100 times with AB-sucrose liquid medium. The resulting solutions were incubated in a 28 °C for 4 days. At the junction of the liquid surface and the air, there was a layer of film. The suspended matter was gently washed with distilled water, and 0.1% crystal violet was added for dyeing for 30 min. The floating color was washed out by distilled water. Ethanol (95%) was then used to dissolve the stained film, and the absorbance at OD_570_ was measured by spectrophotometer (Yidian Analysis Instrument Co., Ltd., Shanghai, China).

### 2.10. Bioinformatic Analysis

To obtain the information of domains and domain architectures, the protein sequence of Atu0526 was uploaded to the SMART server (http://smart.embl-heidelber.de, accessed on 10 April 2021). The server would conduct the sequence alignments and provide information on the protein domains. To predict its 3D structure, the protein sequence was uploaded to the PHYRE2 server (www.sbg.bio.ic.ac.uk/phyre2, accessed on 14 April 2021). The server calculated and output the 3D structure of the protein.

The molecular docking was performed using AutoDock as previous described by Morris et al. [46]. The 3D structural coordinate of formic acid was downloaded from pubchem compound (https://pubchem.ncbi.nlm.nih.gov, accessed on 15 April 2021). Formic acid was docked on the pocket site using the optimized grid box to generate ten docking poses. The docking pose with the highest score was adopted in this study.

### 2.11. Statistical Analysis

Data were statistically analyzed through Microsoft Office Excel data analysis tool. An unpaired Student’s *t* test was performed to assess the statistical difference between the measurements. A value of *p* < 0.05 was considered statistically significant. Significant differences are indicated by * (*p* < 0.05) or ** (*p* < 0.01).

## 3. Results

### 3.1. Atu0526 Is a Conserved sCache-Type MCP in Agrobacterium

**Figure 1:**
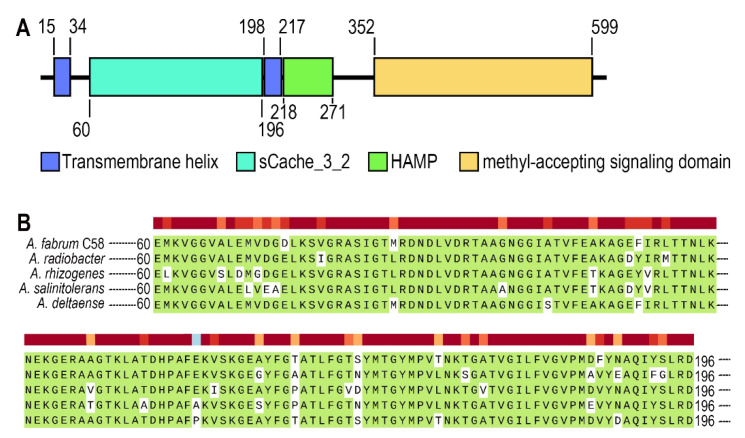
The analysis of Atu0526. (**A**) Domains of Atu0526 predicted by SMART server. The full-length sequence of Atu0526 was input into the SMART server for prediction, and the resulted domains were presented in the linear structure of Atu0526 as rectangles with different colors. The numbers represent the starting and ending positions of each domain. (**B**) sCache domain of Atu0526 was aligned with homologous proteins including Ach5_04520 from *A. radiobacter*, A8L48_17305 from *A. rhizogenes*, AGR9A_Cc210166 from *A. slinitolerans* and AGR7C_Cc260167 from *A. deltaense*. The color above the sequences represents the degree of protein conservation. The green background indicates the identical residues.

Atu0526 is a protein from *A. fabrum* C58 encoded by the gene annotated as *mclA*. It is an HAMP domain-containing protein as described in the National Center for Biotechnology Information (NCBI) Gene database (https://www.ncbi.nlm.nih.gov/gene accessed on 6 July 2021). The domain architecture of Atu0526 is shown in Figure 1A. Atu0526 has two transmembrane helices, a sCache_3_2 domain, an HAMP domain and a methyl-accepting signaling domain (Figure 1A). It is not difficult to conclude that Atu0526 is likely to be a typical sCache MCP with the LBD located in the periplasm.

The sCache domain of Atu0526 was also aligned with those of five homologous MCPs from *A. radiobacter*, *A. rhizogenes*, *A. salinitolerans* and *A. deltaense*. The alignment showed a high similarity among these sequences, suggesting that the proteins are highly conserved in *Agrobacterium* species (Figure 1B). The high degree of conservation may indicate the basic role of the protein in *Agrobacterium*.

### 3.2. Atu0526 Interacts with both CheW1 and CheW2

**Figure 2:**
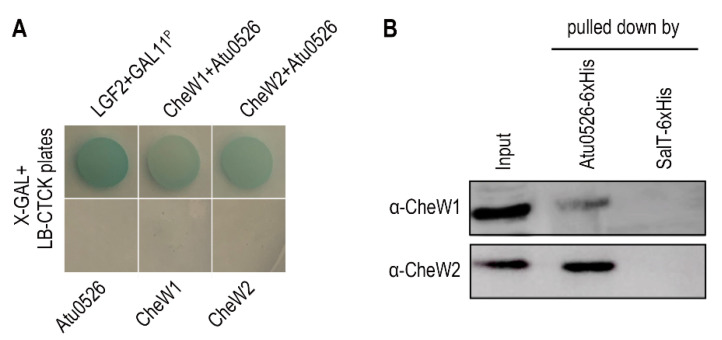
The interaction between Atu0526 and CheWs. (**A**) Bacterial two-hybrid for testing the interactions between Atu0526 and CheWs. The bacterial cells harboring positive control proteins or different combinations of Atu0526 and CheWs were spotted on LB-CTCK plates with X-GAL. Bacterial cells can grow on the plate and appear blue, indicating an interaction between proteins. (**B**) Pull-down assay for testing the interactions between Atu0526 and CheWs. The 6×His-tagged Atu0526 and SalT were incubated with the crude extraction of *A. fabrum* C58 and then pulled down by Ni-IDA Resins. The eluted solutions were subsequently immunoblotted with the antibodies against two CheWs [42] Original blot images in Supplementary Figures S1 and S2.

The interaction with the CheW proteins, as a basic characteristic of the functional MCP, was also tested through bacterial two-hybrid and pull-down experiments. *A. fabrum* C58 has two CheW proteins, CheW1 and CheW2. The bacterial two-hybrid and pull-down experiments confirmed the interaction between Atu0526 and CheW1, and Atu0526 and CheW2 (Figure 2A,B).

### 3.3. LBD of Atu0526 Binds Formic Acid

**Figure 3:**
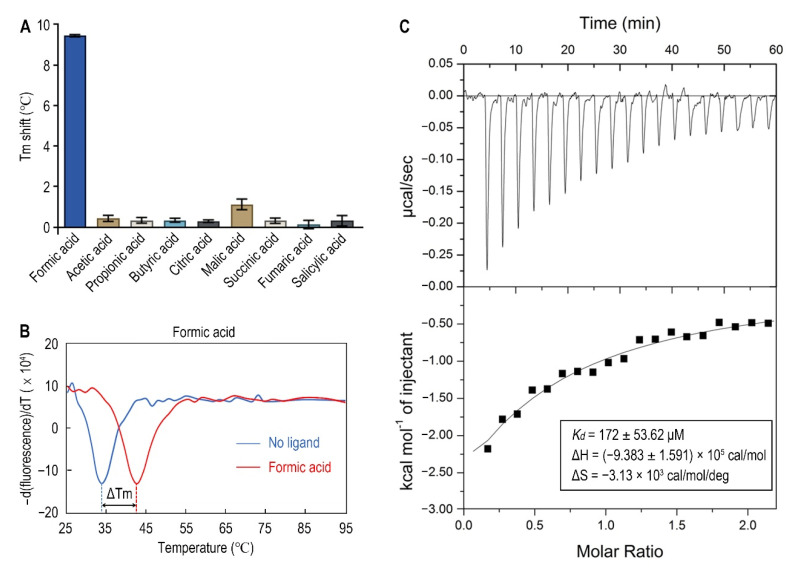
The binding of formic acid to Atu0526^LBD^. (**A**) Thermo shift assay of different organic acids. The column values represent the T_m_ of Atu0526^LBD^ with the corresponding ligands minus the T_m_ without ligand. Column values are the means of three biological replicates with the SD. (**B**) The binding analysis of formic acid to Atu0526^LBD^ using a thermo shift assay. The data are presented in the form of changes in the derivative of fluorescence intensity with temperature. The temperature corresponding to the red and blue dashed lines are the Tm values of Atu0526 with and without formic acid, respectively. (**C**) Isothermal titration calorimetry of Atu0526^LBD^ with formic acid. The upper panel depicts the raw titration data. The lower panel is the isotherm derived by integrating peaks from the raw data and the calculated values of *K_d_*, ∆H and ∆S. Data points were fitted with the “one binding site model” of ORIGIN.

Since organic acids are the major known ligands for sCache receptors [20], nine organic acids were tested as candidates of Atu0526 ligands using a thermo shift assay. As the results showed, the melting temperature (T_m_) showed different shift amplitudes in the presence of different ligands (Figure 3A). Generally, a T_m_ shift of more than 2 °C induced by a ligand is considered significant [44]. The T_m_ of Atu0526^LBD^ was 33.40 ± 0.62 °C while the T_m_ of Atu0526^LBD^ with formic acid added reached 42.42 ± 0.41 °C (Figure 3B). This significant T_m_ shift means that the addition of formic acid greatly enhanced the stability of Atu0526^LBD^, indicating a strong binding of formic acid to the protein. Meanwhile, other organic acids showed no significant T_m_ shift, suggesting a specificity of Atu0526 in binding to formic acid.

In order to obtain direct evidence of the binding between formic acid and Atu0526^LBD^, isothermal titration calorimetry (ITC) was applied. The results confirmed the binding, resulting in an exothermic reaction with a *K_d_* of 172 ± 53.62 μM (Figure 3C). The enthalpy change (ΔH) for the reaction was (−9.383 ± 1.591) ×10^5^ cal/mol, and the entropy change (ΔS) for the reaction was −3.13 × 10^3^ cal/mol/deg (Figure 3C). According to Gibbs free energy calculations (ΔG = ΔH − TΔS), the reaction was spontaneous. Moreover, the values of ΔH and ΔS were all negative, suggesting that hydrogen bond could be the main driving force in this binding reaction.

### 3.4. Formic Acid Is a Chemoattractant of A. fabrum C58

**Figure 4:**
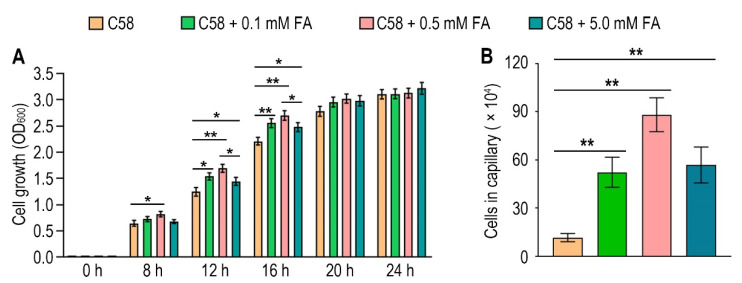
Formic acid can promote the growth of *A. fabrum* C58 and act as its chemoattractant. (**A**) Formic acid promoted the growth of *A. fabrum* C58. To the initial bacterial culture with OD_600_ = 0.03, 0, 0.1 mM, 0.5 mM or 5 mM formic acid were added. OD_600_ of each culture was measured after incubation for 8, 12, 16, 20 and 24 h. (**B**) Capillary assay of *A. fabrum* C58 for formic acid. In the capillary assay, 0, 0.1 mM, 0.5 mM and 5 mM formic acid were applied. Data are the means of three biological replicates with the SD in (**A**) and ten biological replicates with the SD in (**B**). Significant difference (*p* < 0.05) is indicated by * and significant difference (*p* < 0.01) is indicated by ** in (**A**,**B**).

To identify the possible effect of formic acid on *A. fabrum*, we first tested the growth of *A. fabrum* C58 in the presence of different concentrations of formic acid. Contrary to the situation in some other bacteria, the growth of *A. fabrum* C58 was significantly (*p* < 0.05) promoted in the presence of formic acid (Figure 4A), implying that formic acid is beneficial to *A. fabrum* growth and may be a potential chemoattractant of *A. fabrum*. To verify whether *A. fabrum* could be attracted by formic acid, we tested the chemotactic response of *A. fabrum* to different concentrations of formic acid by using a capillary assay. Our results confirmed that *A. fabrum* C58 was significantly (*p* < 0.01) chemoattracted by formic acid. The bacterial population chemoattracted in the capillary tube containing 0.5 mM formic acid was 7.6 times that in the capillary tube without formic acid (Figure 4B).

### 3.5. Atu0526 Is the Only Chemotactic Receptor of A. fabrum C58 to Recognize Formic Acid

**Figure 5:**
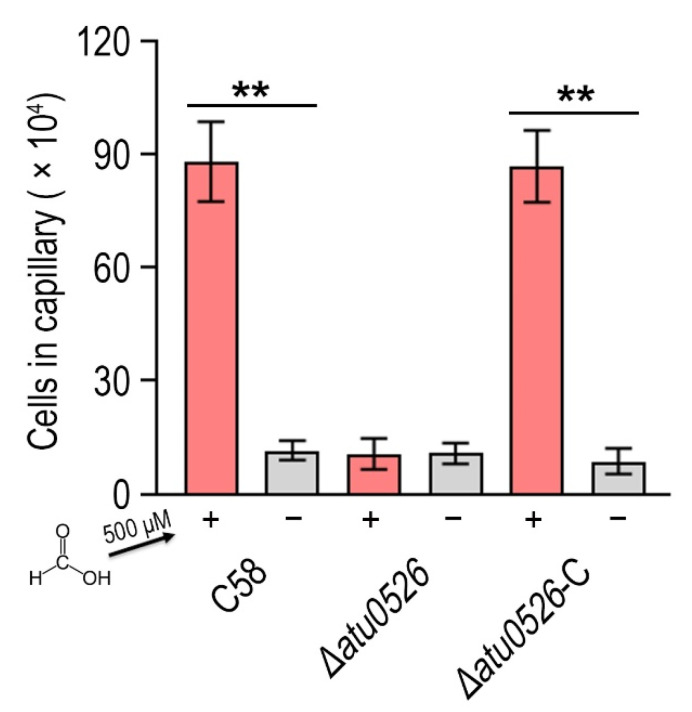
The effect of *atu0526* on the chemotaxis of *A. fabrum* toward formic acid. *A. fabrum* C58, *∆atu0526*, *∆atu0526*-C were tested in a capillary assay with 500 µM formic acid. + Represents 500 µM formic acid was applied, while—represents the case without formic acid. Data are the means of ten biological replicates with the SD. Significant differences (*p* < 0.01) are indicated by **.

Now it has been confirmed that *A. fabrum* C58 is chemoattracted by formic acid and Atu0526^LBD^ can bind formic acid, we needed to confirm whether Atu0526 was the chemoreceptor regulating the chemotaxis of *A. fabrum* toward formic acid. To demonstrate the role of Atu0526 in regulating *A. fabrum* chemotaxis, we constructed two *A. fabrum* derivatives, *atu0526*-deficient mutant (*∆atu0526*) and the complementary strain of *∆atu0526* (*Δatu0526*-C). The Capillary assay showed that the chemotaxis of *A. fabrum* C58 toward formic acid was completely abolished after *atu0526* was knocked out (Figure 5), suggesting that Atu0526 is the only chemoreceptor for formic acid in *A. fabrum* C58. Additionally, the complementary strain *Δatu0526*-C showed the same chemotactic phenotype for formic acid as the wild type strain (Figure 5). Combined with the previous results of the in vitro binding experiments, it can be concluded that Atu05256 is a specific chemoreceptor for formic acid.

### 3.6. Replacement of R115 by Alanine Significantly Increases the Stability of Atu0526^LBD^ and the Affinity to Formic Acid

**Figure 6:**
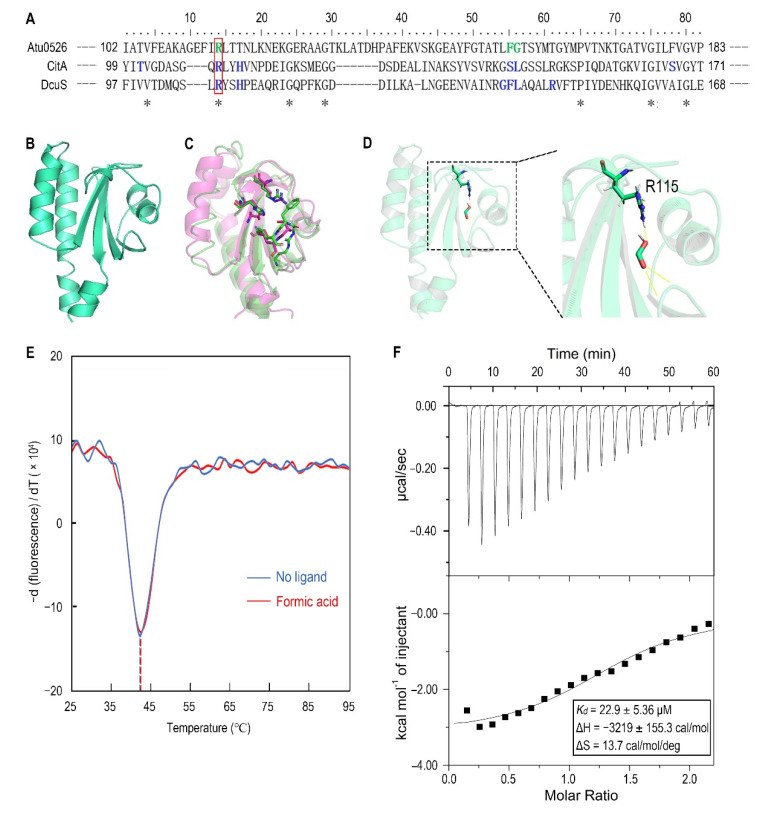
The analysis of R115 on the binding of formic acid to Atu0526^LBD^. (**A**) Sequence alignment of sCache_3_2 domains of Atu0526, CitA and DcuS. * refers to identical residues. The residues shown in blue are residues of CitA and DcuS ligand binding. The residues shown in green were predicted to bind formic acid. The red rectangle indicates R115. (**B**) The predicted 3D structure of sCache_3_2 domain of Atu0526. (**C**) The 3D structures of sCache domains from CitA (magenta, 1P0Z) and DcuS (green, 3BY8). The important residues that ligands bind to are shown in stick form. (**D**) The docking of formic acid with Atu0526^LBD^. The right part is an enlarged presentation of the content in the dashed box. The hydrogen bonds between the ligand and the protein are represented by yellow dashed lines. The R115 is in the stick diagram. (**E**) The binding analysis of formic acid to Atu0526^LBD^R115A using a thermo shift assay. The data are presented in the form of changes in the derivative of fluorescence intensity with temperature. The temperatures corresponding to the red and blue dashed lines are the T_m_ values of Atu0526^LBD^R115A with and without formic acid, respectively. (**F**) Isothermal titration calorimetry of Atu0526^LBD^R115A with formic acid. The upper panel depicts the raw titration data. The lower panel is the isotherm derived by integrating peaks from the raw data and the calculated values of *K_d_*, ∆H and ∆S. Data points were fitted with the “one binding site model” of ORIGIN.

To identify the amino acid residues that are directly involved in the binding of Atu0526^LBD^ to formic acid, the Atu0526 sCache_3_2 domain was aligned with the corresponding domains of the two typical sCache-type receptors, CitA from *Klebsiella pneumoniae* and DcuS from *E. coli* (Figure 6A). Both CitA and DcuS are transmembrane sensors containing a sCache_3_2 domain like Atu0526. The structures of CitA and DcuS LBD domains with ligands have all been resolved [47,48]. The important residues of CitA and DcuS at the interface between proteins and ligands were compared with those of Atu0526. It was noted that the residues of CitA and DcuS that bind ligands are relatively conserved in certain regions (Figure 6A,C). Among these residues, only the arginine at position 115 of Atu0526 was identical to the corresponding residues of CitA and DcuS, suggesting that this residue may play an essential role in the binding. To further scrutinize the residues related to ligand binding, the 3D structure of Atu0526^LBD^ was predicted by the PHYRE2 server, and this protein structure was then interrogated for a possible interaction with formic acid using molecular dynamics modeling. As shown in the docking model, formic acid might form hydrogen bonds with R115, F156 (phenylalanine at position 156) and G157 (glycine at position 157) (Figure 6D). These residues are in the same ligands binding regions as CitA and DcuS (Figure 6A), and it once again hinted that R115 may be important for ligand binding. However, it should be pointed out that there may be other binding-related residues that could not be identified in the predicted model due to the lack of resolved crystal structure.

For R115, the atom predicted to form a hydrogen bond with formic acid is provided by the side group of R115 (Figure 6D). Therefore, we replaced this arginine with alanine to remove the potential force. This single-residue mutated Atu0526^LBD^ variant was designated as Atu0526^LBD^R115A. For F156 and G157, the atoms predicted to form hydrogen bonds with formic acid are provided by the peptide bonds of F156 and G157, not by the side groups. A single-residue mutation to F156 or G157 has no potential significance for the binding analysis. Thus, our study focused on R115. The purified Atu0526^LBD^R115A was then tested in thermo shift assay. The T_m_ of Atu0526^LBD^R115A was 42.19 ± 0.39 °C which was much higher than that of Atu0526^LBD^ (Figure 3B and Figure 6E), indicating that the single-residue mutation increased the protein stability. However, the T_m_ of Atu0526^LBD^R115A only rose by 0.24 °C to 42.43 ± 0.32 °C after adding formic acid (Figure 6E). That was to say, the addition of formic acid did not change the stability of Atu0526^LBD^R115A significantly.

Since adding formic acid did not change the stability of Atu0526^LBD^R115A, did this mean that formic acid would not bind to Atu0526^LBD^R115A? With this question in mind, we conducted an isothermal titration calorimetry to test the binding of formic acid to Atu0526^LBD^R115A. As the results showed, formic acid could bind to Atu0526^LBD^R115A with a *K_d_* of 22.9 ± 5.36 μM (Figure 6F). The *K_d_* was significantly (*p* < 0.01) lower than that of formic acid binding to Atu0526^LBD^, which means Atu0526^LBD^R115A has a stronger affinity with formic acid than Atu0526^LBD^. Although we did not initially expect that Atu0526^LBD^R115A would bind to formic acid, the binding of formic acid to Atu0526^LBD^ had indeed been changed due to the mutation.

### 3.7. Replacement of R115 by Alanine Completely Destroys the Function of Atu0526 in Regulating Chemotaxis, but Not the Cellular Localization

**Figure 7:**
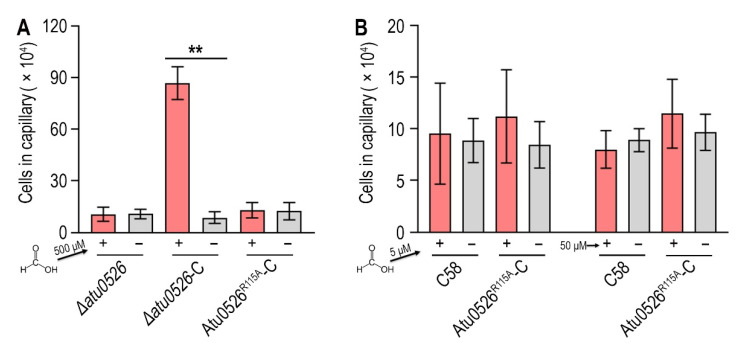
The effect of R115 on the chemotaxis of *A. fabrum* C58 toward formic acid. (**A**,**B**) Capillary assay of strains for formic acid. In (**A**), *∆atu0526*, *∆atu0526*-C and Atu0526^R115A^-C were tested in capillary assay with 500 µM formic acid. In (**B**), *A. fabrum* C58 and Atu0526^R115A^-C were tested with 5 µM and 50 µM formic acid. + Represents 5, 50, or 500 µM formic acid was applied, while − represents the case without formic acid. Data in (**A**,**B**) are the means of ten biological replicates with the SD. Significant differences (*p* < 0.01) are indicated by **.

In order to study whether the binding of formic acid to Atu0526^LBD^R115A can correctly trigger a chemotactic response, we introduced the R115A mutation into *∆atu0526*-C and conducted a capillary assay to assess the chemotactic motility toward formic acid. The results showed that the strain with R115_Atu0526_ replaced by alanine (Atu0526^R115A^-C) was no longer chemotactic toward 500 μM formic acid (Figure 7A). The phenotype of Atu0526^R115A^-C was consistent with *Δatu0526* (Figure 7A). That means that although formic acid can bind to Atu0526^LBD^R115A, this binding does not result an effective chemotactic response at a formic acid concentration of 500 μM.

Since Atu0526^LBD^R115A has a stronger affinity with formic acid, we reduced the formic acid concentration by one and two orders of magnitude in the capillary assay to test if Atu0526^R115A^ could sense a lower concentration of formic acid. The results showed that neither 5 nor 50 μM formic acid could trigger the chemotactic response of Atu0526^R115A^-C (Figure 7B), suggesting that the R115 mutation does not make a more sensitive chemoreceptor, but just a non-functional chemoreceptor for formic acid.

In order to check the possibility that the R115 mutation affects the protein localization, thereby affecting the chemotaxis toward formic acid, we fused green fluorescent protein (GFP) with Atu0526 and Atu0526^R115A^, and expressed the GFP fused proteins in *∆atu0526*. As shown in the pictures, Atu0526-GFP and Atu0526^R115A^-GFP showed similar localization (Supplementary Materials Figure S3), indicating that R115 does not affect the localization of Atu0526.

### 3.8. A Hypothetical Signal Transduction Model of Atu0526

Formic acid can bind to Atu0526^R115A^ but cannot trigger a chemotactic response of Atu0526^R115A^-C, and the localization of Atu0526 is not altered by the residue substitution. Therefore, we suspect that R115 plays a role in the signal transduction of Atu0526. Although the studies of the signal transduction mechanism of sCache-type MCPs are limited, the studies of the non-MCP sCache-type receptors allow to formulate a working hypothesis. The resolved structures of citrate-free and citrate-bound CitA indicate a presence of signal generating mechanism [49]. In CitA, a minor loop has a maximum displacement of 13.54 Å due to the binding of citrate, and the displacement drives the adjacent β-sheet to curl toward the center, thus generating the signal (Supplementary Materials Figure S4) [49]. Several other sCache-type receptors and the molecular dynamic modeling predicted Atu0526 also conform to this mechanism [48,50]. In our molecular dynamic modeling, formic acid binds to the R115, F156 and G157 of Atu0526 (Figure 6D). R115 is in the major loop (the loop connecting the first and second β-sheets of Atu0526^LBD^), while F156 and G157 are in the minor loop (the loop connecting the second and third β-sheets of Atu0526^LBD^). The minor loop is generally flexible in the sCache_3_2 domain [47,48,49]. Therefore, we conjecture that formic acid binds to R115, and then F156 and G157 binds to the formic acid, pulling the minor loop toward the major loop. Such a speculative way of ligand-binding would cause the conformational change of Atu0526^LBD^ to generate the signal.

**Figure 8:**
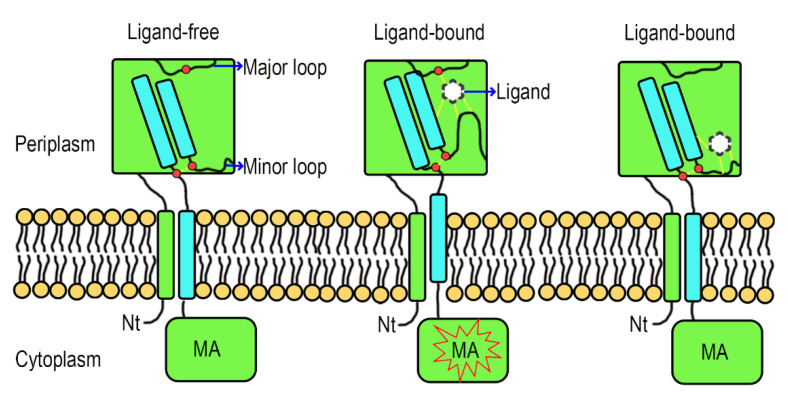
A hypothetical signal transduction model of Atu0526. The formic acid anchors to the anchorage residues (such as R115) and pulls the minor loop upward to realize chemotactic signal transduction. When the anchorage residues are not enough, the ligand may be able to bind to Atu0526, but it is not sufficient to induce a conformational change for chemotactic signal transduction. The red dot above refers to anchorage residue(s), and the two red dots below refer to the C-terminal residues of the fourth β-sheet and the nearby minor loop residues. The force between ligand and Atu0526 or between two residues is indicated by yellow dashed lines.

To make this clear, a hypothetical model showing the signal transduction of Atu0526 was prepared. In this model, when there is no ligand binding to the Atu0526, the minor loop is in a free stretch state, and the C-terminus of the LBD will not form enough movement to induce the MA signaling (Figure 8). When a ligand binds to the LBD correctly, the ligand-binding residues (such as R115) in the major loop act as anchorages that will pull the minor loop closer through the ligand and drive the Atu0526^LBD^ into a closed conformation. This conformational change will cause an upward displacement of the LBD C-terminus, thereby activating the cytoplasmic MA signaling (Figure 8). If there are not enough anchorage residues in the chemoreceptor (Atu0526^R115A^), the ligand may still bind to the LBD. However, such a binding is not able to form a closed conformation and induce MA signaling (Figure 8). This model may explain why Atu0526^R115A^ can bind formic acid but cannot cause a chemotactic response to formic acid.

## 4. Discussion

Although formic acid is broadly used to inhibit bacterial growth, it is experimentally confirmed to be the chemoattractant of *A. fabrum* C58. The ligand concentration used may be a reasonable explanation for this contradiction. The concentration of formic acid used to inhibit bacterial growth generally exceeds 10 mM. Such a concentration is hard to find in natural conditions; therefore, it has little to do with the evolution of the bacterial chemotactic response to formic acid. For *A. fabrum* C58, 500 μM formic acid can promote bacterial growth. Additionally, 5 mM formic acid can also promote the growth of *A. fabrum* C58, but to a lower degree than that of 500 μM formic acid (Figure 4A). It is believed that as the concentration increases, formic acid may still inhibit the growth of *A. fabrum* C58.

It is reported that some chemoreceptors affect the biofilm formation [51,52,53,54,55]. There has also been report of cross-talks between chemotactic systems and biofilm formation, in which CheA may be a common regulator of the two systems [56]. Therefore, we tried to detect the biofilm formation of C58, *Δatu0526*, *Δatu0526*-C and *ΔcheA* (*cheA* mutant in which the entire *cheA* was knocked out). As results showed, with the mutation of *atu0526*, the formation of biofilm was significantly (*p* < 0.01) enhanced, while the mutation of *cheA* reduced the formation of biofilm (Supplementary Materials Figure S5). At present, we still do not know how Atu0526 affect the formation of biofilm, but it is clear that Atu0526 is a negative regulator in the process of biofilm formation. Biofilm formation is usually associated with infection of pathogens [57,58]. Therefore, Atu0526 may affect the infection of *A. fabrum* C58 by affecting biofilm formation. In addition, formic acid is one of the root exudates. Atu0526 as a chemoreceptor of formic acid may also affect the interaction between *A. fabrum* C58 and the hosts by affecting the recognition of the host exudates.

The results of ITC showed that formic acid could bind to Atu0526^LBD^R115A with a higher affinity than that of Atu0526^LBD^ (Figure 3C and Figure 6F). Arginine is an amino acid whose side group can be dissociated, while alanine is an uncharged hydrophobic amino acid. The exchange of these two residues may cause changes of hydrophilicity and ionic environment in the ligand-binding pocket. Such changes are likely to affect the affinity between ligands and receptors [59,60]. Therefore, the higher affinity may be due to the changes in the ligand-binding pocket environment.

In the sCache_3_2 domain, ligand-binding residues are relatively conserved, especially the residue at the corresponding position of R115 (Figure 6A). The docking results suggest that R115 acts as the ligand-binding residue. Our results confirmed the vital role of R115 in the chemotaxis of *A. fabrum* toward formic acid. However, Atu05256^LBD^R115A can still bind to formic acid. Two reasons may explain these results. First, as shown in our hypothetic model, R115 is an anchorage residue. Formic acid can still bind to Atu0526^R115A^, but cannot induce the conformational change by pulling the minor loop. Second, the replacement of R115 with alanine directly caused the T_m_ of Atu0526^LBD^ to increase (Figure 3B and Figure 6E), which means an increase in protein rigidity. The increased protein rigidity due to R115 mutation might make it difficult to produce a conformational change. The entropy change in the ITC experiments can also reflect the conformational stability of Atu0526^LBD^R115A. The ∆S of formic acid binding to Atu0526^LBD^ is −3.13 × 10^3^ cal/mol/deg (Figure 3C). The negative value means that the disorder in the system was reduced after the ligand binding. The reduced disorder indicates a stable state of the ligand-bound protein. On the other hand, the entropy just increased slightly (13.7 cal/mol/deg) after the binding of formic acid to Atu0526^LBD^R115A (Figure 6F). That is to say, the binding did not play a role in reducing the disorder of the system and might not stabilize the conformation of the protein. The conformational stabilization of proteins induced by ligand binding is the principle behind the thermo shift assay [61]. The insignificant Tm shift in the thermal shift assay (Figure 6E) also implies that formic acid might not induce the conformational change of Atu0526^LBD^R115A. Generally, the conformational change of LBD generates a signal. Therefore, R115 may affect signal generation by affecting the conformational change of Atu0526.

The signal transduction mechanism of sCache-type MCPs is still not fully understood. For some 4HB-type MCPs, the final helix is supposed to form a piston-like movement toward the cell membrane after the ligand binding [27]. Crystallographic studies of the non-MCP sCache-type receptor CitA also suggest a piston-like movement after the ligand binding [49]. Based on our molecular dynamic modeling, Atu0526 seems to adopt a similar signal generation mechanism as CitA. Although the sCache domain of CitA and 4HB domain are suggested to form piston-like movement, they move in opposite directions [27,49]. A previous study reported that the chimeric receptor in which sCache-type LBD was fused to the cytosolic fragment of the 4HB-type chemoreceptor could also mediate the response to chemoeffectors [62]. If Atu0526 forms a movement similar to CitA, there will be a problem worthy of further study, that is, how to unify the movement in different directions into the same signal output.

## 5. Conclusions

The broad antibacterial agent formic acid is a chemoattractant of *A. fabrum* C58. Atu0526, a conserved sCache-type chemoreceptor from *A. fabrum* C58, is the only chemoreceptor in *A. fabrum* C58 that regulates chemoattraction toward formic acid. R115 of Atu0526 affects the binding of formic acid to Atu0526 and plays an essential role in Atu0526 regulating the chemotactic response of *A. fabrum* C58 toward formic acid. Our hypothetical model suggests that R115 acts as an anchorage residue to pull the minor loop to the major loop through formic acid. Such a ligand-binding way might cause the conformational change of Atu0526 to generate the signal.

## Data Availability

The data that support the findings of this study are available from the corresponding author upon reasonable request.

## Supplementary Materials

The following are available online at https://www.mdpi.com/2079-7737/10/12/1345/s1, Table S1: Bacterial strains and plasmids used in this study, Table S2: Primers used in this study, Figure S1: Western blotting of the pull-down proteins using CheW1 antibody, Figure S2: Western blotting of the pull-down proteins using CheW2 antibody, Figure S3: Localization of Atu0526-GFP and Atu0526^R115A^-GFP, Figure S4: The structures of periplasmic domain of CitA with and without ligand, Figure S5: Biofilm formation of C58, *Δatu0526*, *Δatu0526*-C and *ΔcheA*.
