# Supplementary material for: Agrobacterium fabrum atu0526-Encoding Protein Is the Only Chemoreceptor That Regulates Chemoattraction toward the Broad Antibacterial Agent Formic Acid
Source: Biology (Basel). 2021 Dec 17;10(12):1345. doi: 10.3390/biology10121345 (PMC8698456; doi:10.3390/biology10121345)
Supplement: Supplementary file 1 [file biology-10-01345-s001.zip › biology-1410860-supplementary.pdf]

Table S1. Bacterial strains and plasmids used in this study.

Strains or Plasmids	Description	Source
**Strains**		
Escherichia coli		
DH5α	*endA1 hsdR17 supE44 thi-1 recA1 gyrA96 relA1 (argF-lacZYA) U169 ϕ80dlacZ.*	Bethesda Research Laboratories
BL21(DE3)	*F⁻ ompT gal dcm lon hsdSB(rB- mB-) λ(DE3 *lacI lacUV5-T7 gene 1 ind1 sam7 nin5])*	[1]
Atu0526LBD	BL21(DE3) strain with pET30a-Atu0526LBD was transformed in.	This study
R115A	BL21(DE3) with pET30a-R115A transformed in.	This study
6×His-Atu0526	BL21(DE3) with the pET30a-6×His-Atu0526 transformed in.	This study
6×His-SalT	BL21(DE3) with the pET30a-6×His-SalT transformed in.	This study
XL1-Blue MR competent cell	*Δ(mcrA)183 Δ(mcrCB-hsdSMR-mrr)173 endA1 supE44 thi-1 recA1 gyrA96 relA1 lac [F´ lacI^q bla lacZ Kan^r]*	BacterioMatch™
Atu0526/CheW1	BacterioMatch™ Two-Hybrid System Reporter Strain with pTRG-CheW1 and pTB-Atu0526 transported in	This study
Atu0526/CheW2	BacterioMatch™ Two-Hybrid System Reporter Strain with pTRG-CheW2 and pTB-Atu0526 transported in	This study
Positive control strain	BacterioMatch™ Two-Hybrid System Reporter Strain with pTRG-GALL^P and pTB-LGF2 transported in	This study
Negative-1	BacterioMatch™ Two-Hybrid System Reporter Strain with pTRG and pTB-Atu0526 transported in	This study
Negative-2	BacterioMatch™ Two-Hybrid System Reporter Strain with pTRG-CheW1 and pTB transported in	This study
Negative-3	BacterioMatch™ Two-Hybrid System Reporter Strain with pTRG-CheW2 and pTB transported in	This study
Agrobacterium fabrum		
C58	Wild-type, nopaline-type pTiC58 plasmid	[2]
△*atu0526*	Derivative of C58 in which *atu0526* was deleted	This study
△*atu0526*-C	Complemented strain of △*atu0526* by *atu0526* gene harbored on plasmid pCB301-*atu0526*	This study
R115A-C	Complemented strain of △*atu0526* by plasmid pCB301-*atu0526*$_{R115A}$, in which the arginine at position 115 was mutated into alanine in the sequence of protein	This study
GFP	△*atu0526* strain with pCB301-*GPF* transformed in.	This study
Atu0526-GFP	Complemented strain of △*atu0526* by *atu0526* fused with *GPF* harbored on plasmid pCB301-*atu0526-GPF*	This study
R115A-GPF	Derivative of Atu0526-GFP strain, in which the arginine at position 115 of Atu0526 was mutated into alanine in the sequence of protein	This study
**Plasmids**		
pEX18Km	Gene replacement vector carrying a counter selectable marker *sacB*, *oriT*, Km^R	[3,4]
pEX18Km-*atu0526*	pEX18Km carrying the upstream and downstream fragments of *atu0526* gene, Km^R; for the deletion of *atu0526* gene	This study

pCB301	A mini binary vector plasmid, with MCS from pBI101, Km[R]	[5]
pCB301-*atu0526*	pCB301 carrying *atu0526* gene and its native promoter sequence, Km[R]	This study
pTRG	pTRG target plasmid in BacterioMatch™ Two-Hybrid System	BacterioMatch™
pTB	pBT bait plasmid in BacterioMatch™ Two-Hybrid System	BacterioMatch™
pTB-Atu0526	pTB plasmid with atu0526 gene inserted	This study
pTRG-CheW1	pTRG plasmid with cheW1 gene inserted	This study
pTRG-CheW2	pTRG plasmid with cheW2 gene inserted	This study
pTRG-GALL[P]	pTRG plasmid with GALL[P] gene inserted	BacterioMatch™
pTB-LGF2	pTB plasmid with LGF2 gene inserted	BacterioMatch™
pET30a	pET30a protein expressing plasmid promoted by the T7 promoter	EMD Biosciences
pET30a-Atu0526LBD	pET30a plasmid with atu0526LBD gene inserted	This study
pET30a-R115A	pET30a plasmid with R115A gene inserted	This study
pET30a-6×His-Atu0526	pET30a plasmid with 6×His-Atu0526 gene inserted	This study
pET30a-6×His-SalT	pET30a plasmid with 6×His-SalT gene inserted	This study

Table S2. Primers used in this study.

**Primers**	**Sequence**	**Description**
atu0526-F1	5'-GCCAGTGCCAAGCTTTATGCGGGGCTGGCTCTGC-3'	To amplify the upstream fragment of *atu0526*
atu0526-R1	5'-GACTTTCAAAAATGGAGAGAGAAACAGTTCCCCAAAGCC-3'	
atu0526-F2	5'-GGCTTTGGGGAACTGTTTCTCTCTCCATTTTTGAAAGTC-3'	To amplify the downstream fragment of *atu0526*
atu0526-R2	5'-GGTACCCGGGGATCCCCGTCTTCATGGCATTCG-3'	
0526C-F	5'-CGCGGATCCCCGTCAATGCCTATCTCGGTTCG-3′	To amplify *atu0526* gene or its single amino acid mutant with its promoter
0526C-R	5'-CCGCTCGAGTCAGGCCGCCCGGTTCAA-3′	
R115A-F	5'-GGTCAGTGCAATGAATTCGCCGGCCTTGG CTTC-3'	To mutate LBD of Atu0526 with arginine at position of 115 replaced by alanine
R115A-R	5'-TTCATTGCACTGACCACCAATCTGAAGAATGAAAAGG-3'	
LBD-F	5'-ATCGGATCCGAATTCTTGAAGGACAACATCATCAC-3'	To amplify *LBD* of *atu0526* or *R115A* for inserting into pET30a
LBD-R	5'-GTGGTGGTGCTCGAGTTACATATCACGCAGGCTGTA-3'	
pET30atu0526-F	5'-ATCGGATCCGAATTCATGCACAACCGGCTCTTTAAGTCC-3'	To amplify full length *atu0526* for inserting into pET30a
pET30atu0526-R	5'-GTGGTGGTGCTCGAGTTATCAGGCCGCCCGGTTCAA-3'	
atu0526-GFP-F1	5'-CGCGGATCCCCGTCAATGCCTATCTCGGTTCG-3′	To amplify *atu0526* or its single amino acid mutant with its promoter for fusing eGFP
atu0526-GFP-R1	5′-CTCGCCCTTGCTCACCATTCAGGCCGCCCGGTTCAA-3′	
atu0526-GFP-F2	5′-TTGAACCGGGCGGCCTGAATGGTGAGCAAGGGCGAG-3′	To amplify *eGFP* for fusing *atu0526* or its single amino acid mutant
atu0526-GFP-R2	5'-CCGCTCGAGTTACTTGTACAGCTCGTCCATGCC-3′	
GFP-F	5'-CGCGGATCCATGGTGAGCAAGGGCGAG-3′	To amplify *eGFP* for inserting into pCB301
GFP-R	5'-CCGCTCGAGTTACTTGTACAGCTCGTCCATGCC-3′	
pTRGatu0526-F	5′-GAGGCGGCCGGATCCATGCACAACCGGCTCTTTAAGTCC-3′	To amplify *atu0526* for inserting into pTRG

pTRGatu0526-R	5′-GCTCAGACTGAATTCTTATCAGGCCGCCCGGTTCAA-3′	
pBTCheW1-F	5′-GGCCGCATCGAATTCATGTCCAACGCCATCAAGCAA-3′	To amplify *cheW1* for inserting into pBT
pBTCheW1-R	5′-TTAACTCGAGGATCCTCAGGCCGCTTCGCGCGCC-3′	
pBTCheW2-F	5′-GGCCGCATCGAATTCCTGATGGCAATGATTAACTC-3′	To amplify *cheW2* for inserting into pBT
pBTCheW2-R	5′-TTAACTCGAGGATCCCAGGCGGCAAGATCTTCGG-3′	
pTRGCheW1-F	5′-GAGGCGGCCGGATCCATGTCCAACGCCATCAAGCAA-3′	To amplify *cheW1* for inserting into pTRG
pTRGCheW1-R	5′-GCTCAGACTGAATTCTCAGGCCGCTTCGCGCGCC-3′	
pTRGCheW2-F	5′-GAGGCGGCCGGATCCCTGATGGCAATGATTAACTC-3′	To amplify *cheW2* for inserting into pTRG
pTRGCheW2-R	5′-GCTCAGACTGAATTCCAGGCGGCAAGATCTTCGG-3′	

The underlines indicate restriction sites.

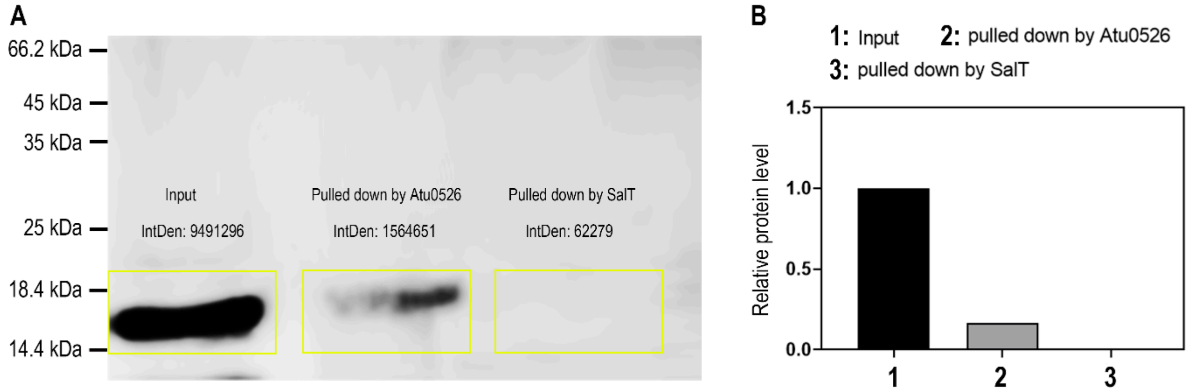

Figure S1. Western blotting of the pull-down proteins using CheW1 antibody. (A) Original blot image of Figure 1C upper part. Lines from left to right are input, 6×His-tagged Atu0526 and 6×His-tagged SalT. The IntDEN is read by ImageJ (National Institutes of Health, Maryland, USA). Prior to the readings, images were converted to grayscale as follows: Image -> Type –> 8 bit, next: Image -> Adjust–> Brightness/Contrast –> Auto. The densitometry/intensity (IntDen) is read from the area of the yellow rectangle. (B) Relative protein level indicated by the densitometry/intensity reading in (A). The value is the ratio of each densitometry/intensity reading to that of the band of "Input" line.

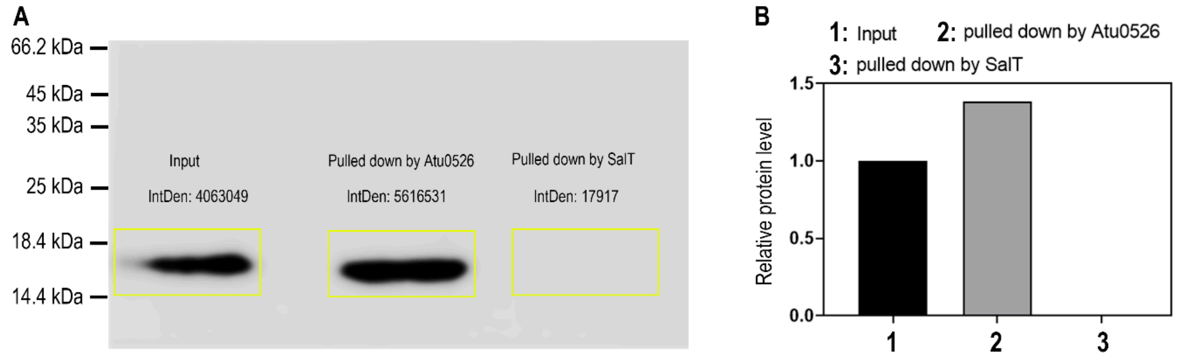

Figure S2. Western blotting of the pull-down proteins using CheW2 antibody. (A) Original blot image of Figure 1C lower part. Lines from left to right are input, 6×His-tagged Atu0526 and 6×His-tagged SalT. The IntDEN is read by ImageJ (National Institutes of Health, Maryland, USA). Prior to the readings, images were converted to grayscale as

follows: Image -> Type –> 8 bit, next: Image -> Adjust–> Brightness/Contrast –> Auto. The densitometry/intensity is read from the area of the yellow rectangle. (B) Relative protein level indicated by the densitometry/intensity reading in (A). The value is the ratio of each densitometry/intensity reading to that of the band of "Input" line.

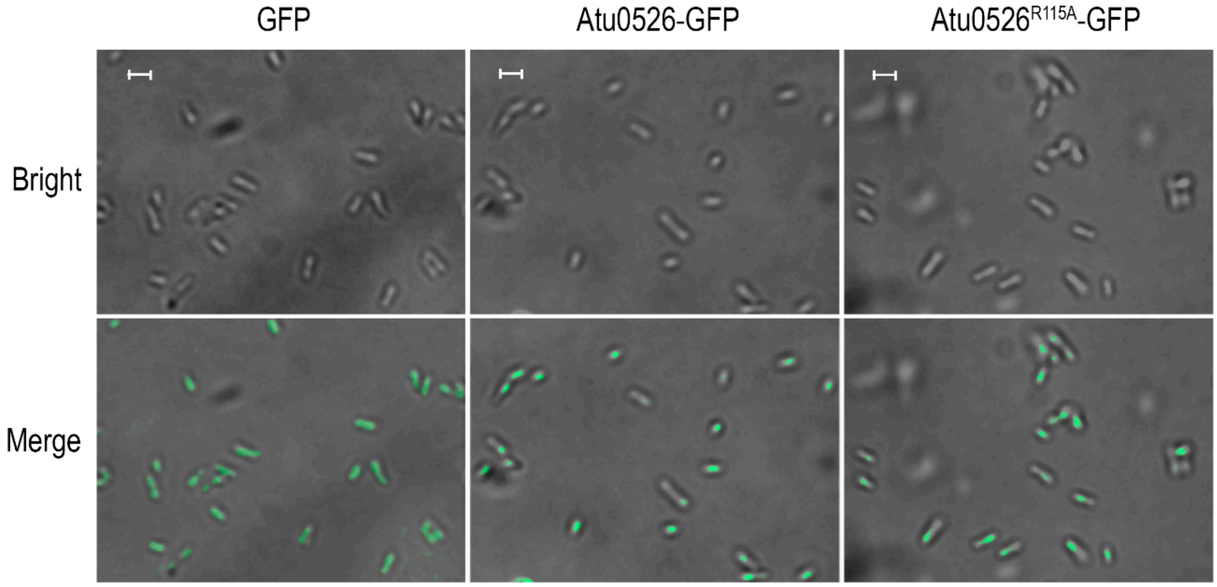

Figure S3. Localization of Atu0526-GFP and Atu0526^{R115A}-GFP. 'Bright' indicates the images taken under the brightfield, and 'Merged' indicates combinations of pictures from fluorescent and bright field. The scale bar on the picture represents a length of 2 μm.

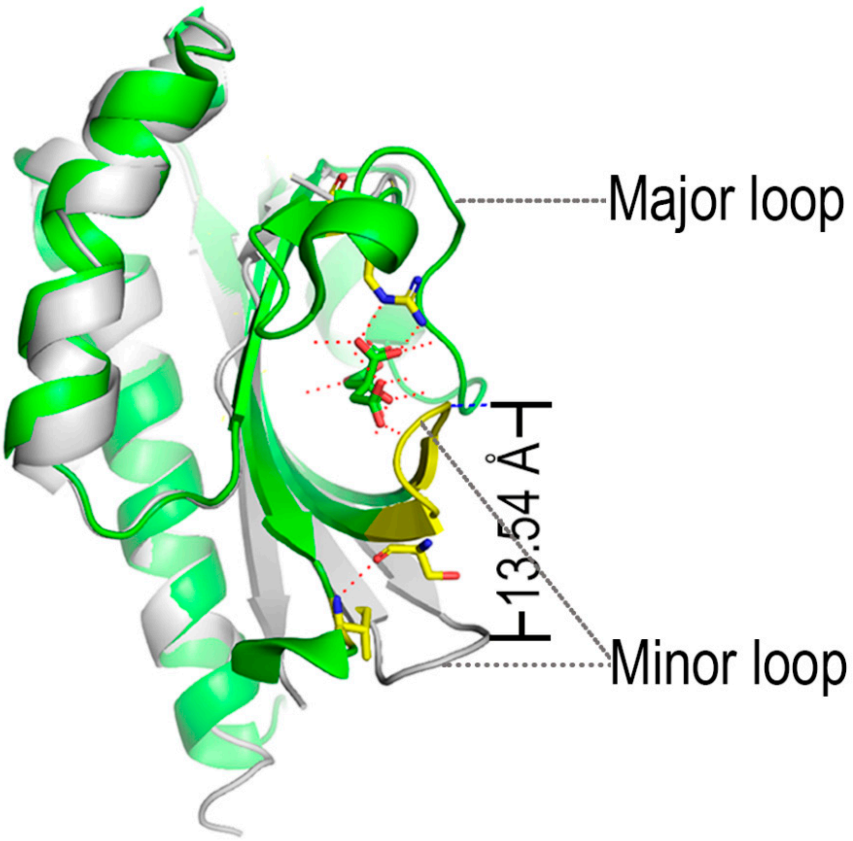

Figure S4. The structures of periplasmic domain of CitA with ligand (Green, 2J80) and without ligand (gray, 2V9A) [51]. The minor loop has a maximum displacement of 13.45 Å in these cases. The hydrogen bonds between the ligand and the protein are represented by red dashed lines, and the

hydrogen bond between the C-terminal residue of the fifth β-sheet and the nearby residue in the minor loop is indicated by a red dashed line. The key arginine residues, minor loops and C-terminal residue of the fifth β-sheet are all shown in yellow.

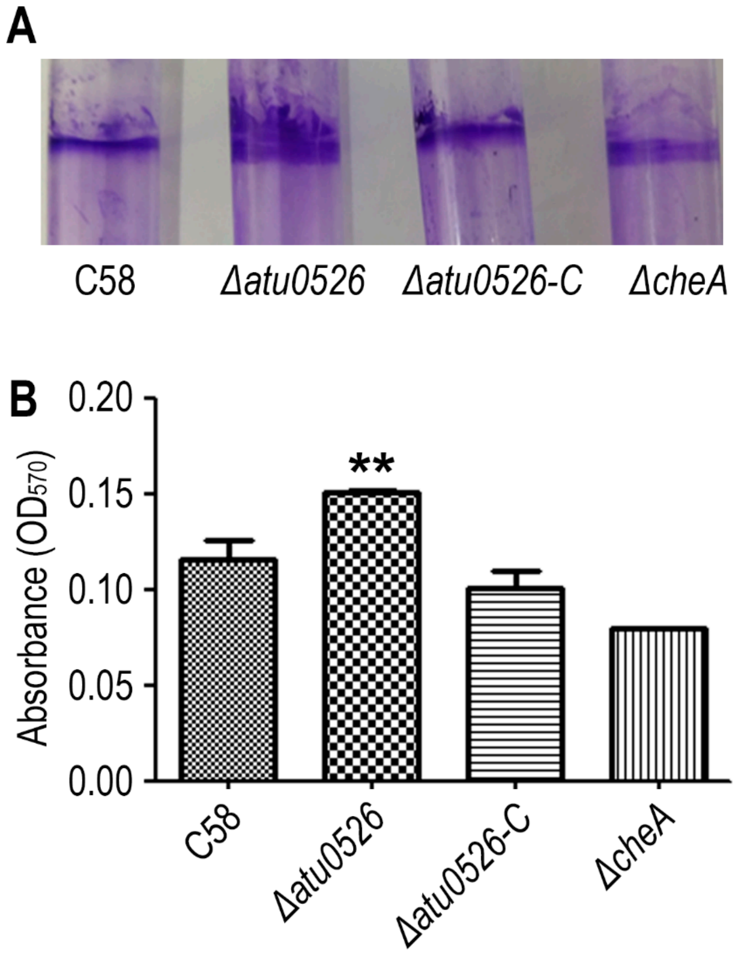

Figure S5. Biofilm formation of C58, *Δatu0526*, *Δatu0526*-C and *ΔcheA*. (A) The bacterial biofilm identified by crystal violet staining. (B) Quantification analysis of biofilm formation of each strain. Significant difference ($p<0.01$) between C58 and *Δatu0526* is indicated by **.

References

1. Studier, F.W.; Moffatt, B.A. Use of bacteriophage T7 RNA polymerase to direct selective high-level expression of cloned genes. *J. Mol. Biol.* **1986**, *189*, 113–130.
2. Thomashow, M.F.; Nutter, R.; Montoya, A.L.; Gordon, M.P.; Nester, E.W. Integration and organization of Ti plasmid sequences in crown gall tumors. *Cell* **1980**, *19*, 729–739.
3. Hoang, T.T.; Karkhoff-Schweizer, R.R.; Kutchma, A.J.; Schweizer, H.P. A broad-host-range Flp-FRT recombination system for sites pecific excision of chromosomally-located DNA sequences: Application for isolation of unmarked Pseudomonas aeruginosa mutants. *Gene* **1998**, *212*, 77–86.
4. Guo, M.; Hou, Q.M.; Hew, C.L.; Pan, S.Q. *Agrobacterium* VirD2-binding protein is involved in tumorigenesis and redundantly encoded in conjugative transfer gene clusters. *Mol. Plant Microbe Interact*. **2007**, *20*, 1201–1212.
5. Xiang, C.; Han, P.; Lutziger, I.; Wang, K.; Oliver, D.J. A mini binary vector series for plant transformation. *Plant Mol. Biol*. **1999**, *40*, 711–717.
